# Amino acid PET vs. RANO MRI for prediction of overall survival in patients with recurrent high grade glioma under bevacizumab therapy

**DOI:** 10.1007/s00259-024-06601-4

**Published:** 2024-01-17

**Authors:** Artem Chaban, Birgit Waschulzik, Denise Bernhardt, Claire Delbridge, Friederike Schmidt-Graf, Arthur Wagner, Benedikt Wiestler, Wolfgang Weber, Igor Yakushev

**Affiliations:** 1 Department of Nuclear Medicine, School of Medicine, Klinikum Rechts der Isar, Technical University of Munich, Munich, Germany; 2 Institute of AI and Informatics in Medicine, School of Medicine, Klinikum Rechts der Isar, Technical University of Munich, Munich, Germany; 3 Department of Radiation Oncology, School of Medicine, Klinikum Rechts der Isar, Technical University of Munich, Munich, Germany; 4 Department of Pathology, School of Medicine, Klinikum Rechts der Isar, Technical University of Munich, Munich, Germany; 5 Department of Neurology, School of Medicine, Klinikum Rechts der Isar, Technical University of Munich, Munich, Germany; 6 Department of Neurosurgery, School of Medicine, Klinikum Rechts der Isar, Technical University of Munich, Munich, Germany; 7 Department of Neuroradiology, School of Medicine, Klinikum Rechts der Isar, Technical University of Munich, Munich, Germany

**Keywords:** Positron emission tomography, Magnetic resonance imaging, Avastin, Pseudoresponse, Therapy monitoring, Glioblastoma

## Abstract

**Purpose:**

To summarize evidence on the comparative value of amino acid (AA) PET and conventional MRI for prediction of overall survival (OS) in patients with recurrent high grade glioma (rHGG) under bevacizumab therapy.

**Methods:**

Medical databases were screened for studies with individual data on OS, follow-up MRI, and PET findings in the same patient. MRI images were assessed according to the RANO criteria. A receiver operating characteristic curve analysis was used to predict OS at 9 months.

**Results:**

Five studies with a total of 72 patients were included. Median OS was significantly lower in the PET-positive than in the PET-negative group. PET findings predicted OS with a pooled sensitivity and specificity of 76% and 71%, respectively. Corresponding values for MRI were 32% and 82%. Area under the curve and sensitivity were significantly higher for PET than for MRI.

**Conclusion:**

For monitoring of patients with rHGG under bevacizumab therapy, AA-PET should be preferred over RANO MRI.

## Introduction

Despite a multimodal treatment, the prognosis of patients with recurrent high-grade glioma (rHGG) remains poor. While a survival benefit of bevacizumab (BEV) has been questionable [1–4], it is considered as a treatment option especially in symptomatic patients with rHGG [5]. Magnetic resonance imaging (MRI) is the imaging modality of choice in glioma patients. Initially developed for clinical trials, assessment in neuro-oncology criteria (RANO) has become a standard in clinical settings, too [6]. Along with the FLAIR changes, dynamics of contrast enhancement is a key component of the RANO criteria. As BEV affects permeability of the blood brain barrier (BBB), reducing contrast enhancement on T1 MRI, assessment of response to the BEV therapy is challenging. Thus, a so-called pseudoresponse is a common phenomenon in this setting [7]. Since amino acid (AA) PET relies on metabolic activity of tumor tissue rather than changes in BBB permeability, it may detect glioma progression in a more sensitive manner than the MRI RANO criteria [8]. Nevertheless, conventional MRI is still widely used in this setting [9, 10]. The aim of this study was to summarize evidence on the *comparative* value of AA-PET and RANO MRI for prediction of recurrent overall survival (OS) in patients with rHGG under BEV therapy.

## Methods

Medical databases MEDLINE, PubMed, EMBASE, and the Cochrane Library were screened for studies in English using various combinations of key words: recurrent high grade glioma, glioblastoma, amino acid PET, therapy response, and bevacizumab. The last search was performed on the 22nd of August 2023. Inclusion criteria were availability of individual data on OS, follow-up MRI as assessed according to the RANO criteria, and follow-up AA-PET, allowing us to relate the imaging findings with OS at 9 months. The follow-up PET was rated according to local institutional criteria as pathological (PET +) or normal (PET −) relative to the baseline PET prior to BEV therapy. To produce the binary outcomes for MRI, we post hoc treated complete response, partial response, and stable disease as normal (MRI −), while progressive disease as pathological (MRI +).

**Fig. 1:**
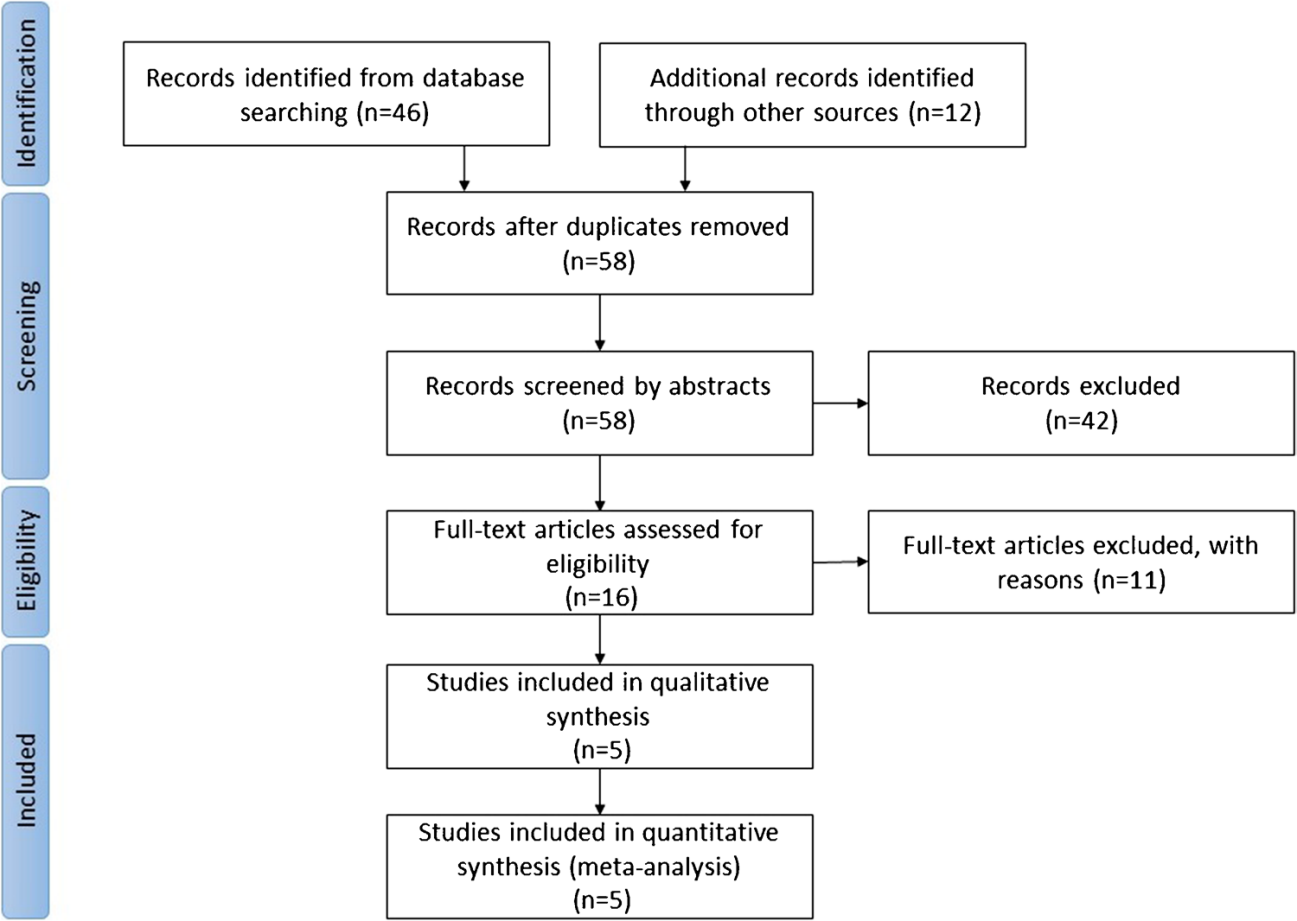
Selection of studies as PRISMA flow diagram

The process of selection of eligible studies is depicted in Fig. 1. Out of 16 full-text articles, 11 were excluded for the following reasons: lack of individual data (*n* = 8), too short OS in relation to the imaging findings (3 and 6 months, *n* = 2), and a tracer other than amino acid (*n* = 1). Thus, 5 studies [11–15] with a total of 72 patients were included (Table 1). Due to a lack of OS data, three patients in [11] and one patient in [12] were excluded. PET and RANO MRI were evaluated in their ability to predict OS at 9 months. The threshold of 9 months was based on two major clinical trials of BEV: in the BELOB trial, an OS at 9 months was used to avoid uncertainties in assessing response and progression under BEV therapy [2]; in the BRAIN trial, a median overall survival of roughly 9 months (9.2 months in the BEV alone group and 8.7 months in the BEV + irinotecan group) was reported [16]. To this end, sensitivity and specificity of each method with corresponding 95% confidence intervals (CI) were calculated using a meta-analysis. McNemar tests were applied to compare sensitivity and specificity between the methods. In addition, we performed a receiver operating characteristic (ROC) analysis. The DeLong test was used to compare areas under the curves (*AUC*). All analyses were conducted two-sided using a 5% level of significance. The statistical analyses were performed using statistical software R as reported previously [17].

**Table 1:** Characteristics of eligible studies

Study	Tracer	Study design	Patients in the present meta-analysis	Imaging system	Concomitant chemotherapy
Hutterer et al. [11]	FET	Retrospective	8	PET/MRI	Irinotecan
Galldiks et al. [12]	FET	Prospective	9	PET/MRI	Irinotecan
Schwarzenberg et al. [14]	FDOPA	Prospective	23	PET	Irinotecan*
Deuschl et al. [15]	MET	Prospective	11	PET/MRI	Lomustine
Galldiks et al. [13]	FET	Prospective	21	PET/MRI	Lomustine

*FET* O-(2-18F-fluoroethyl)-L-tyrosine, *MET* 11C-methyl-L-methionine, *FDOPA* 3,4-dihydroxy-6-[18F]-fluoro-L-phenylalanine

* Three patients in [14] were treated with bevacizumab alone

## Results

**Fig. 2:**
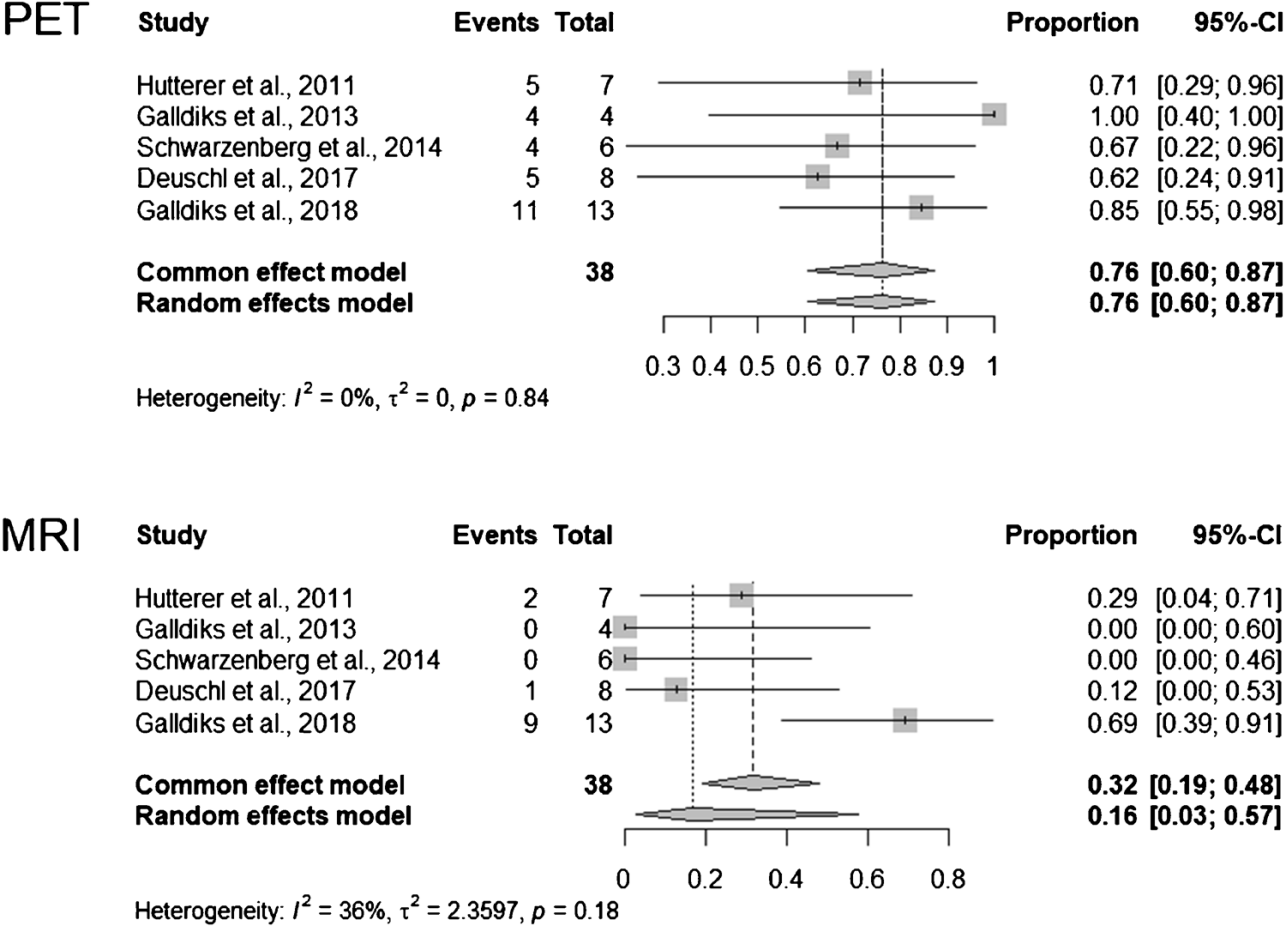
Forest plots for sensitivity. Events column lists the number of true-positives. Total column shows sum of true-positives and false-negatives. Proportion column lists reported sensitivity of individual publications and 95% *CI*. Length of diamonds corresponds to *CI*. Vertical line represents pooled sensitivity

**Fig. 3:**
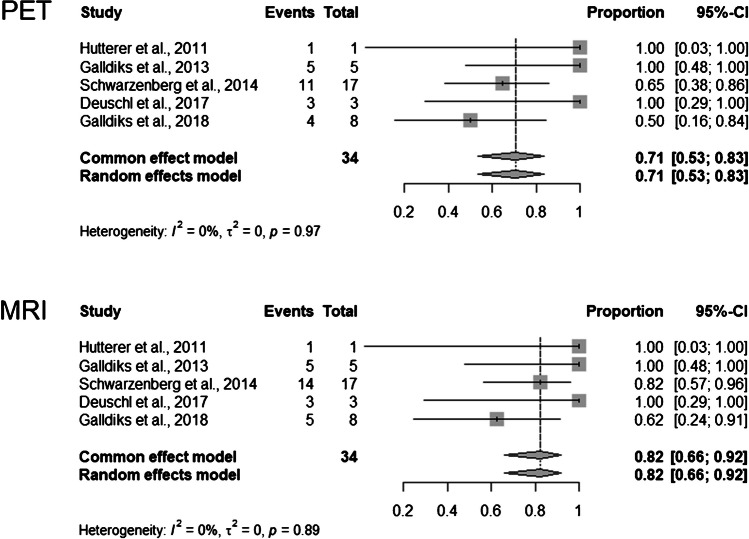
Forest plots for specificity. Events column lists the number of true-negatives. Total column shows sum of true-negatives and false-positives. Proportion column lists reported specificity of individual publications and 95% *CI*. Length of diamonds corresponds to *CI*. Vertical line represents pooled specificity

**Fig. 4:**
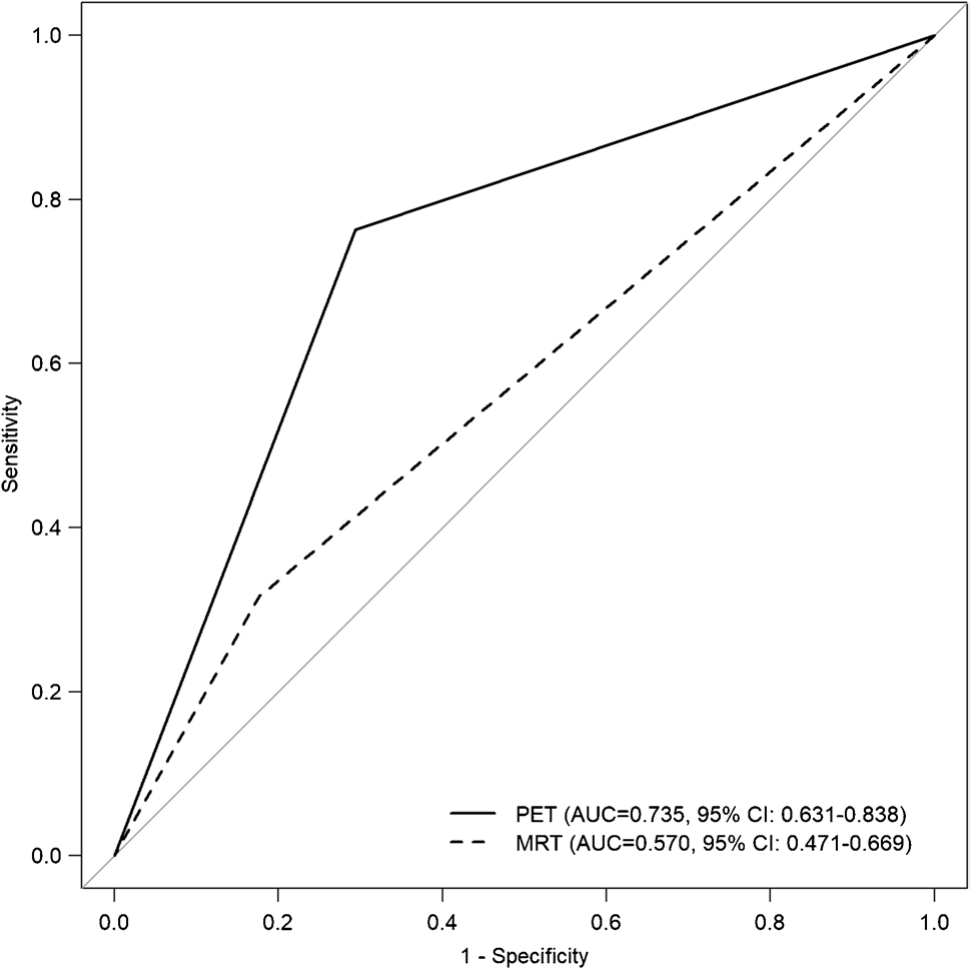
ROC curves for PET and MRI. PET corresponds to the solid line, MRI to the dashed line

The median OS was 8.8 months (range 1.4–38). In the Mann–Whitney *U* test, OS was significantly (*p* < 0.001) lower in the PET + (*median* = 6.1; *n* = 39) than in the PET − (*median* = 12.3; *n* = 33) group. OS was marginally (*p* = 0.052) lower in the MRI + (*median* = 6.8; *n* = 18) than in the MRI − (*median* = 10.5; *n* = 54) group. The PET + findings predicted OS at 9 months with a sensitivity and specificity of 76% (95% *CI* 60–87) and 71% (95% *CI* 53–83), respectively (Figs. 2 and 3). Corresponding values for MRI were 32% (95% *CI* 19–48) and 82% (95% *CI* 66–92) (Figs. 2 and 3). Heterogeneity was overall low (Figs. 2 and 3). The difference between the *AUC*s for PET and MRI was − 0.17 (95% *CI* − 0.04, − 0.29; *p* = 0.007) (Fig. 4). The difference for sensitivity was statistically significant (*p* < 0.001), for specificity it was not (*p* = 0.344).

## Discussion

This is the first meta-analysis on the comparative value of AA-PET and RANO MRI for prediction of OS in patients with rHGG under BEV therapy. PET was found to predict OS at 9 months with a significantly higher sensitivity, while specificity did not differ between the methods. An important strength of this study is availability of PET and MRI data in the same patients at the same time point, allowing a direct comparison of the methods.

Pooled sensitivity of AA-PET and RANO MRI was found to be 76% and 32%, respectively. Obviously, sensitivity of 76% is far from perfect. Still, it is more than twice of sensitivity of MRI. Given the anti-angiogenic effect of BEV, poor sensitivity of RANO MRI is not unexpected. Nevertheless, the RANO criteria are still commonly used in monitoring of patients with rHGG under BEV therapy [9, 10]. Our results clearly argue that with sensitivity below that by chance, the value of conventional MRI in this setting is very limited.

Pooled specificity did not significantly differ between AA-PET (71%) and conventional MRI (82%). In our study specificity refers to the ability to correctly identify patients without the disease progression. Given that BEV reduces permeability of the BBB, high specificity of RANO MRI is not unexpected. Notably, advanced MRI, such as dynamic contrast-enhanced MRI, can detect progressive disease under BEV therapy more accurately [18]. Somewhat lower specificity of PET can be explained by local inflammatory processes, e.g., reactive astrocytosis, after radiation and systemic therapy, which may result in tracer uptake above the level of normal brain tissue [19].

This study has certain limitations. First, the included studies applied different PET criteria, such as a reduction in metabolic tumor volume and tumor-to-normal brain ratio. Second, the diagnosis of HGG in the included studies was based on older diagnostic criteria and did not take into account mixed HGG pathology. Furthermore, the low number of the eligible studies did not allow sub-analyses according to the IDH mutation status and concomitant chemotherapy.

In sum, this work provides a high level evidence on the superior value of AA-PET relative to RANO MRI for prediction of OS in patients with rHGG under BEV therapy.

## Data Availability

Data are available upon request.
